# A Comprehensive Assessment of ^68^Ga-PSMA-11 PET in Biochemically Recurrent Prostate Cancer: Results from a Prospective Multicenter Study on 2,005 Patients

**DOI:** 10.2967/jnumed.121.262412

**Published:** 2022-04

**Authors:** Monica Abghari-Gerst, Wesley R. Armstrong, Kathleen Nguyen, Jeremie Calais, Johannes Czernin, David Lin, Namasvi Jariwala, Melissa Rodnick, Thomas A. Hope, Jason Hearn, Jeffrey S. Montgomery, Ajjai Alva, Zachery R. Reichert, Daniel E. Spratt, Timothy D. Johnson, Peter J.H. Scott, Morand Piert

**Affiliations:** 1 Radiology Department, University of Michigan, Ann Arbor, Michigan;; 2 Ahmanson Translational Theranostics Division, Department of Molecular and Medical Pharmacology, UCLA, Los Angeles, California;; 3 Department of Radiology and Biomedical Imaging, University of California San Francisco, San Francisco, California;; 4 Department of Radiation Oncology, University of Michigan, Ann Arbor, Michigan;; 5 Urology Department, University of Michigan, Ann Arbor, Michigan;; 6 Internal Medicine Department, University of Michigan, Ann Arbor, Michigan; and; 7 Department of Biostatistics, University of Michigan, Ann Arbor, Michigan

**Keywords:** prostate cancer, prostate-specific membrane antigen, PSMA, disease pattern, hybrid PET

## Abstract

We prospectively investigated the performance of the prostate-specific membrane antigen (PSMA) ligand ^68^Ga-PSMA-11 for detecting prostate adenocarcinoma in patients with elevated levels of prostate-specific antigen (PSA) after initial therapy. **Methods:** ^68^Ga-PSMA-11 hybrid PET was performed on 2,005 patients at the time of biochemically recurrent prostate cancer after radical prostatectomy (RP) (50.8%), definitive radiation therapy (RT) (19.7%), or RP with postoperative RT (PORT) (29.6%). The presence of prostate cancer was assessed qualitatively (detection rate = positivity rate) and quantitatively on a per-patient and per-region basis, creating a disease burden estimate from the presence or absence of local (prostate/prostate bed), nodal (N1: pelvis), and distant metastatic (M1: distant soft tissue and bone) disease. The primary study endpoint was the positive predictive value (PPV) of ^68^Ga-PSMA-11 PET/CT confirmed by histopathology. **Results:** After RP, the scan detection rate increased significantly with rising PSA level (44.8% at PSA < 0.25%–96.2% at PSA > 10 ng/mL; *P* < 0.001). The detection rate significantly increased with rising PSA level in each individual region, overall disease burden, prior androgen deprivation, clinical T-stage, and Gleason grading from the RP specimen (*P* < 0.001). After RT, the detection rate for in-gland prostate recurrence was 64.0%, compared with 20.6% prostate bed recurrence after RP and 13.3% after PORT. PSMA-positive pelvic nodal disease was detected in 42.7% after RP, 40.8% after PORT, and 38.8% after RT. In patients with histopathologic validation, the PPV per patient was 0.82 (146/179). The SUV_max_ of histologically proven true-positive lesions was significantly higher than that of false-positive lesions (median, 11.0 [interquartile range, 6.3–22.2] vs. 5.1 [interquartile range, 2.2–7.4]; *P* < 0.001). **Conclusion:** We confirmed a high PPV for ^68^Ga-PSMA-11 PET in biochemical recurrence and the PSA level as the main predictor of scan positivity.

## Visual Abstract

**Figure:**
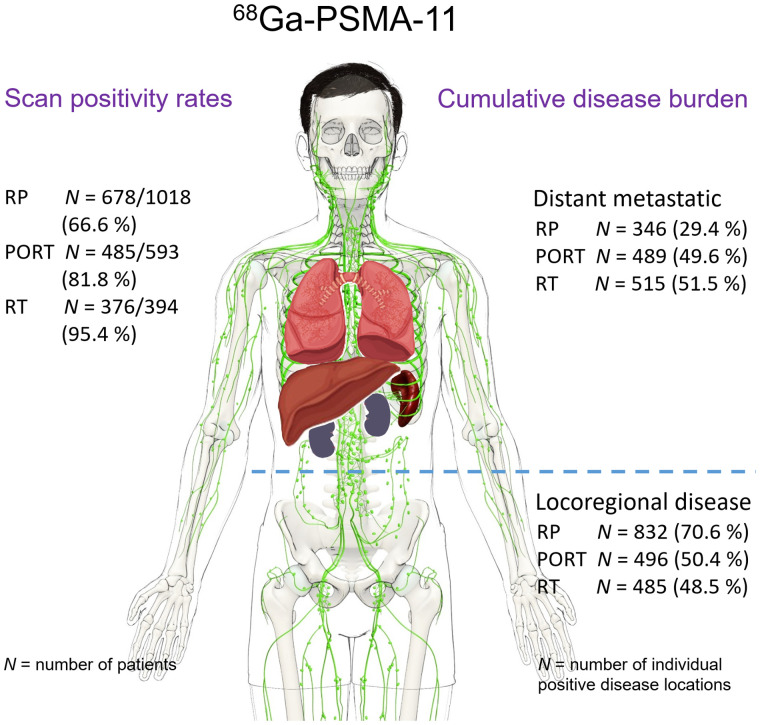

Biochemical recurrence (BCR) is an independent risk factor in survival outcomes (*1*) after radical prostatectomy (RP) and radiation therapy (RT) for localized prostate cancer. BCR after definitive therapy is common, especially in higher-risk disease, and may affect more than 50% of patients over the long term (*2*,*3*).

With broadened use of newer prostate-specific membrane antigen (PSMA)–based radioligands to identify the location of prostate cancer using PET, the treatment of BCR is rapidly changing to more personalized and targeted approaches (*4*). Although a large body of retrospective evidence is available suggesting that ^68^Ga-PSMA-11 has high accuracy (*5*,*6*), prospective studies that include gold-standard histologic verification are rare (*7*). The difficulty in obtaining pathologic confirmation of PSMA PET–positive (suggestive) lesions is related to the high positivity rate at a relatively low disease burden and the challenges in sampling small and difficult-to-reach lesions.

To better comprehensively assess the performance characteristics of ^68^Ga-PSMA-11 PET, 3 institutions—the University of Michigan, UCLA, and the University of California San Francisco—combined their prospective trial datasets of patient populations with BCR disease to determine the accuracy of ^68^Ga-PSMA-11 based on histopathology and to identify predictors of PET positivity and patterns of recurrence.

## MATERIALS AND METHODS

### Patients

The Food and Drug Administration granted the use of ^68^Ga-PSMA-11 under 3 investigational-new-drug applications. Imaging was performed within registered prospective clinical studies assessing the diagnostic performance of ^68^Ga-PSMA-11 PET in BCR of prostate cancer at the University of Michigan (ClinicalTrials.gov NCT03396874), UCLA (NCT02940262), and the University of California San Francisco (NCT03803475). The respective Institutional Review Boards of each institution approved these study protocols. From February 2018 to December 2020, 2,005 patients were enrolled with histologically proven prostate cancer and BCR after RP with or without postoperative RT (PORT) (prostate-specific antigen [PSA] level > 0.2 ng/mL, >6 wk after surgery) or definitive RT (PSA nadir + ≥2 ng/mL). Patients with another active malignancy within the last 2 y (excluding skin basal cell or cutaneous superficial squamous cell carcinoma that has not metastasized and superficial bladder cancer) were not eligible. Patients who received any interval treatment other than androgen deprivation therapy (ADT) were excluded. Prior conventional imaging was not required for study participation. All subjects provided written informed consent.

### ^68^Ga-PSMA-11 PET

The investigational radiotracer ^68^Ga-PSMA-11 was manufactured as described in the literature either from generator-produced ^68^Ga-GaCl_3_ (*8*,*9*) or from cyclotron production of ^68^Ga via a liquid target and a GE Healthcare FASTlab synthesis module (*10*). Imaging was performed on dedicated hybrid PET/CT (*n* = 1886) or PET/MRI (*n* = 119) scanners according to a standardized imaging protocol (*8*). On average, 61 min (median, 60 min; interquartile range [IQR], 57–65 min) after intravenous administration of 203.5 MBq (5.5 mCi) (IQR, 185–229 MBq [5.0–6.2 mCi]) of ^68^Ga-PSMA-11, a static emission scan was performed from the thighs to the vertex. A time-of-flight acquisition was performed in 936 of 2,005 (47%) scans. Images were reconstructed using iterative ordered-subset expectation maximization according to vendor recommendations. UCLA performed a diagnostic 2.5-mm collimation CT scan (200–240 mAs, 120 kV) with intravenous contrast medium on either a Siemens Biograph 64 TruePoint or a Siemens Biograph mCT scanner. At the University of Michigan, CT scans (3-mm collimation) were either low-dose (100 mAs, 120 kV on a Biograph 6 TruePoint) or dose-modulated (Biograph mCT) without intravenous contrast medium. The University of California San Francisco investigators performed PET/CT (GE Healthcare Discovery, Biograph mCT, or Philips Vereos scanners) or PET/MRI at 2.5-mm collimation, dependent on scanner availability and contraindications. Diagnostic CT was performed with a standard protocol (80–100 mA, 120 kV) before the PET scan, with intravenous contrast medium for most scans (*7*). All imaging devices received American College of Radiology accreditation.

### Image Data

^68^Ga-PSMA-11 scans were analyzed locally at each institution according to recent guidelines (*8*) by experienced nuclear medicine physicians with access to clinical information, histopathology results, and prior imaging studies when available. Any focal ^68^Ga-PSMA-11 uptake above location-specific background levels was considered PSMA-positive.

**FIGURE 1.**
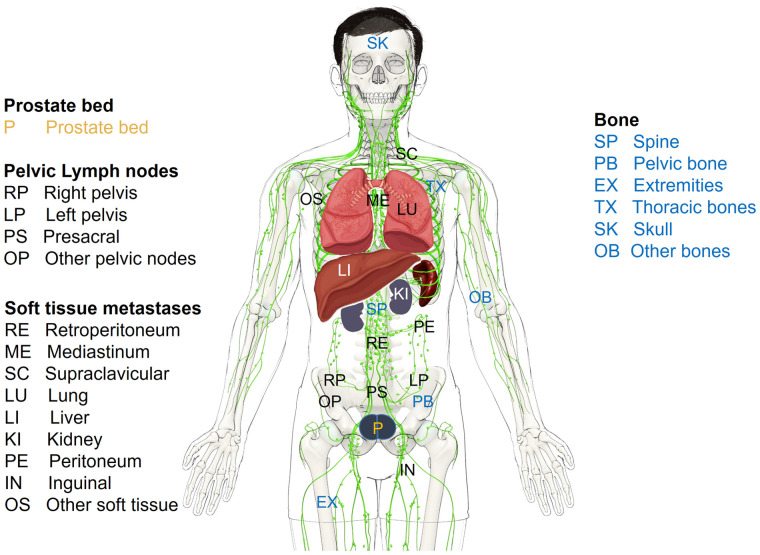
Schematic of identified PSMA-positive lesion locations.

The presence of prostate cancer was quantitatively assessed on a per-region (prostate/prostate bed, pelvis, soft tissue, bone) and per-patient basis. Each involved region was added to estimate the total disease burden (0 = no disease, 1–4 = sum of positive regions). Figure 1 indicates the sampled specific disease locations among these 4 regions.

### Lesion Validation and Quantification

Lesion validation was based on histopathologic analysis only. For lesions with histopathologic verification, the SUV_max_ was obtained. When the lesion could be identified with a clear margin on anatomic imaging (either CT or MRI), the maximum lesion diameter was also recorded.

### Statistical Analysis

The positive predictive value (PPV) of ^68^Ga-PSMA-11 PET/CT confirmed by histopathology was the primary study endpoint. Receiver-operating-characteristic analysis was used to determine the optimal SUV_max_ threshold to separate benign from malignant foci. For contingency table analyses, χ^2^ tests were used to assess hypotheses. For continuous data, the Wilcoxon rank-sum test was applied. Logistic regression was used to study the association between clinically relevant disease parameters, scan characteristics, and scan positivity rates.

## RESULTS

### Patient Characteristics

Table 1 displays the characteristics of 2,005 enrolled subjects with BCR of prostate cancer after RP (51.2%), PORT (29.4%), or definitive RT (19.4%). Age, initial PSA level, PSA nadir, clinical T-stage, and T-stage from the RP specimen were similar among groups. As shown in Supplemental Table 1 (supplemental materials are available at http://jnm.snmjournals.org), Gleason grade groups derived from 1,586 RP specimens did not significantly differ between RP- and PORT-treated patients (*P* = 0.1). However, the Gleason grade groups at biopsy were significantly higher in the 389 patients receiving RT than in the 481 RP patients (*P* < 0.001).

**TABLE 1.** Patient Characteristics

Characteristic	RP (*n* = 1,018 [51.2%])	PORT (*n* = 593 [29.4%])	RT (*n* = 394 [19.4%])
Site (*n*)			
University of Michigan	372 (36.5%)	264 (44.5%)	192 (48.7%)
University of California San Francisco	109 (10.7%)	82 (13.8%)	81 (20.6%)
UCLA	537 (52.8%)	247 (41.7%)	121 (30.7%)
Age (y)			
Median	68 (IQR, 63–73)	70 (IQR, 65–74)	72 (IQR, 67–77)
No. missing	0	0	0
PSA at scan (ng/mL)			
Median	0.78 (IQR, 0.4–2.3)	1.58 (IQR, 0.7–3.3)	5.7 (IQR, 3.4–10.9)
No. missing	0	0	0
PSA nadir (ng/mL)			
Median	0.2 (IQR, <0.1–0.3)	<0.1 (IQR, <0.1–0.7)	0.3 (IQR, 0.1–0.8)
No. missing	458	212	46
Initial PSA at diagnosis (ng/mL)			
Median	7 (IQR, 5.1–11.3)	6.4 (IQR, 4.7–10.2)	7.6 (IQR, 5.4–7.6)
No. missing	651	336	209
T-stage (RP)			
Median	8 (IQR, 6–10)	7 (IQR, 6–10)	NA
No. missing	230	433	
Clinical T-stage			
Median	3 (IQR, 3–4)	3 (IQR, 3–4)	3 (IQR, 3–6)
No. missing	19	433	126
Gleason grade group (RP)			
Median	3 (IQR, 2–5)	3 (IQR, 2–5)	NA
No. missing	19	5	
Gleason grade group at biopsy			
Median	3 (IQR, 2–4)	3 (IQR, 2–4)	3 (IQR, 2–4)
No. missing	727	403	5
PSMA injected dose (MBq)			
Median	200 (IQR, 185--229)	204 (IQR, 185--229)	215 (IQR, 185--233)
No. missing	4	3	1
Treatment-to-scan interval (mo)			
Median	40.2 (IQR, 8.4–104.0)	82.3 (IQR, 48.4–130.6)	75.4 (IQR, 40.3–122.5)
No. missing	23	15	24

NA = not applicable.

T-stage nomenclature: T1 = 1, T1a = 2, T1b = 3, T1c = 4, T2 = 5, T2a = 6, T2b = 7, T2c = 8, T3 = 9, T3a = 10, T3b = 11, T4 = 12.

Most patients treated by RP and PORT had no treatment between the initial therapy and the scan (83.0%), whereas 50.2% of RT-treated patients had received ADT. The interval between the initial therapy and the scan was significantly shorter for patients with persistent PSA after RP or PORT (18.1 mo; IQR, 4.5–72.4 mo) than for patients who achieved undetectable PSA levels after RP or PORT (75.0 mo; IQR, 41.0–125.5 mo) (*P* < 0.001).

### Detection Rate

Given the differences in PSA entry criteria after definitive RT, scan detection rates for RT below a PSA level of 2.0 ng/mL were not available. The scan positivity rate was 78.0% for the entire population but was not evenly distributed among treatment groups (*n* = 712 [67.1%] for RP; *n* = 590 [83.1%] for PORT; *n* = 422 [95.9%] for RT).

Table 2 displays the detection rate for all 3 therapy groups categorized by PSA ranges. A significant increase in the detection rate with rising PSA level was seen for RP-treated (*P* < 0.001) and PORT-treated (*P* < 0.001) patients. In a subcohort of 777 patients treated by RP with lower PSA levels (<1.0 ng/mL) at the time of the scan, the detection rate was significantly higher in PORT-treated (*n* = 208; 71.6%) than in RP-treated (*n* = 569; 52.7%) patients (*P* < 0.001). In the same subcohort, the detection rate was also higher in patients with interval ADT (71.2%) than in those without (54.4%, *P* < 0.02). The detection rate was positively correlated with the Gleason grade groups obtained from the RP specimen (*n* = 1,586 [999 RP and 587 PORT]; *P* < 0.001) and with clinical T-stage (*n* = 670 [242 RP, 160 PORT, and 242 RT]; *P* < 0.01) but not with Gleason grade groups at the initial prostate biopsy (*n* = 870 [291 RP, 190 PORT, and 289 RT]; *P* = 0.86).

**TABLE 2.** ^68^Ga-PSMA-11 Per-Patient Detection Rate Stratified by PSA Level and Prior Therapy

	Total			RP			PORT			RT		
PSA range (ng/mL)	No. Neg.	No. Pos.	% Pos.	No. Neg.	No. Pos.	% Pos.	No. Neg.	No. Pos.	% Pos.	No. Neg.	No. Pos.	% Pos.
<0.25	64	52	44.8	61	44	41.9	3	8	72.7			
0.25–<0.5	160	163	50.5	138	120	46.5	22	43	66.2			
0.5–<1.0	104	234	69.2	70	136	66.0	34	98	74.2			
1.0–<2.0	66	235	78.1	40	128	76.2	26	107	80.5			
2.0–<5.0	46	414	90.0	18	120	87.0	19	131	87.3	9	163	94.8
5.0–<10.0	18	238	93.0	10	74	88.1	3	55	94.8	5	109	95.6
≥10	8	203	96.2	3	56	94.9	1	43	97.7	4	104	96.3
Total	466	1539	76.8	340	678	66.6	108	485	81.8	18	376	95.4

Neg. = negative; Pos. = positive.

Given the high detection rate for patient treated by RT (roughly 95% at any PSA range), no significant relationship was found between PSA and detection rate (Table 2). When the entire patient population was considered (*n* = 2,005), the regional detection rates for local failure (prostate or prostate bed) (Supplemental Table 2; *P* < 0.001), pelvic nodal disease (Supplemental Table 3; *P* < 0.001), distant metastatic disease in soft tissue (Supplemental Table 4; *P* < 0.001), and bone (Supplemental Table 5; *P* < 0.001) increased significantly with PSA level.

### Lesion Validation per Histopathology

Supplemental Table 6 summarizes the patient characteristics of positive scans with (*n* = 179) or without (*n* = 1,360) histopathologic analyses of PSMA-positive lesions. Risk parameters (PSA, PSA nadir, clinical T-stage, Gleason grade groups, locoregional and distant metastatic disease extent) and scan parameters were similar among groups. The average lesion-based PPV was 0.82 for the entire histopathologically assessed population (Table 3). Tissue samples were obtained by either needle biopsy (75%) or surgical resection (25%). Given varying accessibility and risk of specific lesion locations, the number of samples obtained decreased from prostate gland/prostate bed (43%) to soft tissues (26%), pelvic lymph nodes (24%), and bone (7%). The region-specific PPV increased from 0.72 in pelvic lymph nodes to 0.83 in prostate/prostate bed and bone and to 0.88 in soft-tissue lesions. Most false-positive (FP) lesions (*n* = 33) were noted in the prostate region, including 10 foci (after RT) in the prostate gland and 4 lesions in the prostate bed, as well as 1 in a seminal vesicle. Other common locations were pelvic lymph nodes (*n* = 9) and soft-tissue lesions (*n* = 8), including extrapelvic lymph nodes or masses, inguinal lymph nodes, and 1 benign neoplasm (Supplemental Table 7).

**TABLE 3.** ^68^Ga-PSMA-11 Accuracy Confirmed by Histopathology per Region

	All groups combined				RP				PORT				RT			
Site	*n*	TP	FP	PPV	*n*	TP	FP	PPV	*n*	TP	FP	PPV	*n*	TP	FP	PPV
Prostate and prostate bed	77	64	13	0.83	10	8	2	0.80	7	6	1	0.86	60	50	10	0.83
Pelvic lymph nodes	47	34	13	0.72	25	16	9	0.64	19	16	3	0.84	3	2	1	0.67
Soft tissue	43	38	5	0.88	7	5	2	0.71	26	25	1	0.96	10	8	2	0.80
Bone	12	10	2	0.83	2	2	0	1.00	4	3	1	0.75	6	5	1	0.83
Total	179	146	33	0.82	44	31	13	0.80	56	50	6	0.89	79	65	14	0.82

On a per-patient basis, the available SUV_max_ was significantly higher for the 141 true-positive (TP) lesions (median, 11.0; IQR, 6.3–22.2) than for the 30 FP lesions (median, 5.1; IQR, 2.2–7.4) (*P* < 0.001), whereas the maximum size of lesions was similar between groups (TP, 1.3 [IQR, 0.8–2.13]; FP, 1.05 [IQR, 0.75–2.13]). Receiver-operating-characteristic analysis as a function of lesion SUV_max_ resulted in an area under the receiver-operating-characteristic curve of 0.77. At the optimal SUV_max_ threshold (7.5) for differentiating malignant from benign findings, the sensitivity was 69% and the specificity was 80%.

### Disease Burden and Pattern

As shown in Supplemental Table 8, involvement of a single region was the most common outcome except for patients with a PSA level of at least 10 ng/mL. Nonetheless, the rate of multiregion involvement increased steadily with rising PSA level in the entire patient population, as we saw for each individual treatment group (RP, PORT, and RT) (*P* < 0.001).

**FIGURE 2.**
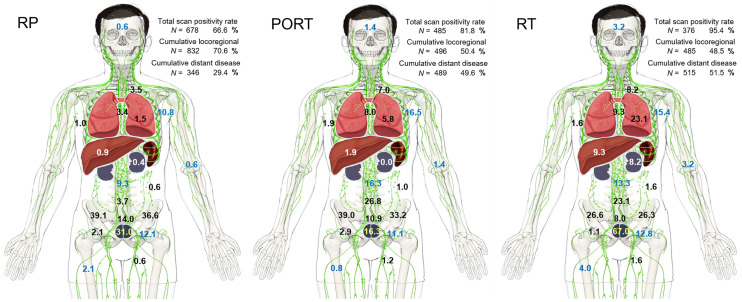
Cumulative total scan positivity rate (relative to entire population in treatment groups RP [*n* = 1,018], RP and PORT [*n* = 593], or definitive RT [*n* = 394]). Percentage of individual PSMA-positive disease locations as listed in Figure 1 and cumulative locoregional and distant disease positivity rates are from PSMA scans rated positive (RP, *n* = 678; PORT, *n* = 485; RT, *n* = 376).

Figure 2 displays the rate of observed disease at encountered locations indicating differences among treatment groups. The supplemental videos highlight the rising disease burden at each region and location per PSA level. After RT, the most likely positive single region was the prostate (252/394, 64.0%), whereas nodal metastatic disease was the predominant location for RP (435/1,018, 42.7%) and PORT (301/593, 50.8%). Among pelvic lymph nodes, predominant locations were central pelvic nodes (internal/external/common iliac, including obturator), followed by presacral and all other pelvic nodal stations. The probability of bilateral disease involvement increased with PSA level in all 3 treatment groups (RP and RT, *P* < 0.001; PORT, *P* < 0.05). When considering only patients with PSA levels of at least 2 ng/mL, the rate of locoregional disease (prostate/bed or pelvic lymph nodes) was similar among groups (Supplemental Table 9).

As shown in Supplemental Table 10, distant metastatic disease increased with rising PSA level, though it was unequally distributed among treatment groups and disease locations (soft tissue vs. bone). Soft-tissue metastases (Supplemental Table 4), including the frequently encountered retroperitoneal lymph node metastases, were found more often after PORT (188/593, 31.7%) than after RP (154/1,018, 15.1%) at any PSA level. Similar results were noted for osseous metastases (PORT, 162/593 [27.3%], vs. RP, 162/1,018 [15.9%]) (Supplemental Table 5). When osseous lesions was detected, the most common locations were pelvic, thoracic, and spinal.

## DISCUSSION

As reviewed recently in a large, retrospective cohort (*11*) and prior metaanalyses (*6*,*12*), a substantial body of evidence exists to support the use of ^68^Ga-PSMA-11 in BCR of prostate cancer. However, this evidence is derived mostly from retrospective studies, and prospective data are rare (*7*).

We present the largest prospectively obtained population of patients with BCR of prostate cancer undergoing ^68^Ga-PSMA-11 PET after initial therapy with curative intent. It comprises the highest number of histologically confirmed PSMA-positive lesions and the largest prospective dataset assessing scan detection rates, particularly at the most relevant PSA range below 1.0 ng/mL after RP and cases of biochemical failure after primary RT.

The primary endpoint of the study was the PPV and not the sensitivity and specificity of the test. This approach was required because histopathologic verification is typically obtained only from PSMA-positive lesions. By limiting the analysis of diagnostic efficacy to histopathologically proven PSMA-positive lesions, we avoided uncertainties related to less stringent clinical endpoints, often referred to as composite endpoints.

The results indicated a high PPV similar to other prospectively obtained data obtained with ^68^Ga-PSMA-11 (*7*) and ^18^F-DCFPyL (*13*), although slightly lower than retrospective single-center data, as recently reviewed (*14*). The discrepancy may be related to differences in patient populations with unknown proportions of sampling errors. Furthermore, tissue sampling is often obtained from equivocal findings. Histopathologic sampling was overrepresented after RT compared with RP or PORT. In contrast to Fendler et al. (*15*), the probability of FP results was similar in all treatment groups and thus not elevated in the setting of prostate lesions after RT. In our cohort, FP assessments were more likely with a lower SUV_max_ whereas the average lesion size of FP and TP lesions was comparable, indicating that simple sampling errors (due to smaller lesion size) could not explain a higher FP rate of low-uptake lesions. However, the individual reader threshold to define positive lesions may have influenced the probability of a TP outcome. Although we cannot exclude this possibility, interreader agreement with ^68^Ga-PSMA-11 is generally high (*16*). Although the selected SUV_max_ threshold of 7.5 differentiated malignant from benign findings with moderate sensitivity and specificity, the large overlap between the SUV_max_ of TP and FP lesions limits the ability of this threshold to reliably predict prostate cancer.

A large body of evidence exists to support a strong relationship between lesion detection rates and PSA levels in BCR of prostate cancer (*7*). Our data show a scan positivity rate of 44.8% at a PSA level of below 0.25 ng/mL and 50.5% for a PSA level of between 0.25 and 0.5 ng/mL for patients after RP. These data are in line with the literature obtained from retrospective analyses (*11*,*12*,*17*). The correlation of PSMA-scan positivity and PSA level has relevant clinical implications. First, it is well established that the success of salvage RT (SRT) after RP is related to the pre-SRT PSA level (*18*). Patients with a high pre-SRT PSA level (>2 ng/mL) have very high rates of recurrence after SRT. In contrast, in patients with PSA levels close to 0.2 ng/mL, more than 75% treated with SRT have long-term durable tumor control (*19*). Additionally, the finding that the pattern of spread after RP with a rising PSA level demonstrates increased nodal, distant, and multiregion disease further helps explain the findings from Radiation Therapy Oncology Group trial 9601. This trial showed a large overall survival benefit from the addition of hormone therapy at PSA levels of more than 1.5 ng/mL after RP but no improvement in metastases or survival for patients treated with early SRT (*20*). This finding may be due to the benefit of ADT in patients with metastatic disease, and men with a presalvage PSA level of more than 1.5 ng/mL have a high probability of already harboring regional and distant metastatic disease.

Prior conventional imaging (CT of abdomen or pelvis and bone scans) was not required for participation in this study, mainly because conventional imaging is often noncontributory in biochemically recurrent prostate cancer and therefore is increasingly omitted as part of the standard of care (*16*,*21*). However, since available prior conventional imaging was allowed to contribute to scan interpretations, such information may have been a potential source of bias.

In our patient population, we noted substantial differences in the pattern of PSMA-positive disease across PSA ranges and treatment groups, as highlighted in the supplemental videos. However, these differences may be based wholly on a pronounced selection bias. We emphasize that the risk of recurrent prostate cancer and the location and extent of metastatic disease are dependent on many factors not assessed in this trial. Confounding factors include differences in risk at the time of diagnosis, heterogeneity and interval advances in therapeutic techniques within treatment groups, and variations in disease management after initial therapy. Furthermore, the observed rate of recurrent and metastatic disease per region in each treatment group does not provide information about the overall rate of recurrent prostate cancer, as scans were performed exclusively on patients expected to present with recurrent disease. Nonetheless, the study offers insight about the relationship of initial risk factors (Gleason score, initial PSA, PSA nadir, age), interval treatment (ADT), and PSA outcome at the time of the scan with ^68^Ga-PSMA-11 scan findings.

## CONCLUSION

Our prospective multicenter trial confirmed that ^68^Ga-PSMA-11 PET is an accurate and effective modality to identify BCR of prostate cancer. Our data indicate a specific recurrent disease pattern for initial therapy approaches and PSA ranges. Half of all scans performed at PSA levels below 0.5 ng/mL had positive results, opening the door for PSMA-targeted focal therapy approaches at an early time point of disease recurrence. Although knowledge of the disease location is of great importance for SRT planning, it remains to be seen whether PSMA image–guided (focal) therapy of BCR of prostate cancer can improve outcomes.

## DISCLOSURE

Jeremie Calais reports prior consulting activities for Advanced Accelerator Applications, Blue Earth Diagnostics, Curium Pharma, GE Healthcare, EXINI, IBA RadioPharma, Janssen Pharmaceuticals, Lantheus, POINT Biopharma, Progenics, Radiomedix, and Telix Pharmaceuticals outside the submitted work. Daniel Spratt received personal fees from Janssen, AstraZeneca, Blue Earth, and Boston Scientific and funding from Janssen. Morand Piert reports prior consulting activities for Bayer and received grant funding from Endocyte/Novartis, Blue Earth Diagnostics, Progenics, the Michigan Prostate SPORE (NIH/NCI 5P50CA186786), and the Department of Radiology of the University of Michigan. Peter Scott received funding from the Department of Radiology of the University of Michigan and grants from Endocyte/Novartis and GE Healthcare. Thomas Hope received grant funding from the National Cancer Institute (R01CA212148 and R01CA235741), the Prostate Cancer Foundation (2017 Young Investigator Award and 2019 VAlor Challenge Award 18CHAL03), Advanced Accelerator Applications, and Philips and served as a consultant for Ipsen, Blue Earth Diagnostics, and Curium. Zachery Reichert received personal fees from AstraZeneca and Dendreon and grant funding from AstraZeneca, the Prostate Cancer Foundation (2018 Young Investigator Award), and the Michigan Prostate SPORE (NIH/NCI 5P50CA186786). No other potential conflict of interest relevant to this article was reported.

## Notes

KEY POINTS **QUESTION:** What is the diagnostic efficacy of ^68^Ga-PSMA-11 in BCR of prostate cancer? **PERTINENT FINDINGS:** Compared with histopathology, ^68^Ga-PSMA-11 PET provides a high PPV to identify biochemically recurrent prostate cancer locoregionally and in distant metastases. The scan detection rate increases with PSA level and is PSA-dependent within individual regions. Higher detection rates are noted with increasing overall disease burden, prior ADT, higher clinical T-stage, and Gleason grade group ratings after RP. **IMPLICATIONS FOR PATIENT CARE:** ^68^Ga-PSMA-11 PET provides a high diagnostic value in BCR of prostate cancer.
